# Evolution and emergence of infectious diseases in theoretical and real-world networks

**DOI:** 10.1038/ncomms7101

**Published:** 2015-01-16

**Authors:** Gabriel E. Leventhal, Alison L. Hill, Martin A. Nowak, Sebastian Bonhoeffer

**Affiliations:** 1 Institute for Integrative Biology, ETH Zürich, 8092 Zürich, Switzerland; 2 Program for Evolutionary Dynamics, Departments of Mathematics and Organismic and Evolutionary Biology, Harvard University, Cambridge, Massachusetts 02138, USA

## Abstract

One of the most important advancements in theoretical epidemiology has been the development of methods that account for realistic host population structure. The central finding is that heterogeneity in contact networks, such as the presence of ‘superspreaders’, accelerates infectious disease spread in real epidemics. Disease control is also complicated by the continuous evolution of pathogens in response to changing environments and medical interventions. It remains unclear, however, how population structure influences these adaptive processes. Here we examine the evolution of infectious disease in empirical and theoretical networks. We show that the heterogeneity in contact structure, which facilitates the spread of a single disease, surprisingly renders a resident strain more resilient to invasion by new variants. Our results suggest that many host contact structures suppress invasion of new strains and may slow disease adaptation. These findings are important to the natural history of disease evolution and the spread of drug-resistant strains.

## Abstract (web summary)

The continual evolution of pathogens makes infectious disease control very challenging. Here the authors examine how host population structure influences disease evolution, and show that heterogeneous contact networks lower the fixation probability of newly arising pathogen strains.

## Graphical abstract

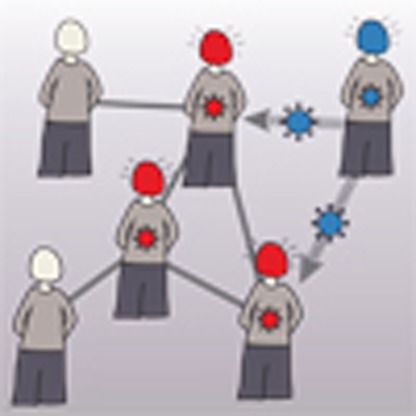

Our ability to understand and control the spread of infectious diseases has historically relied on insights gained through mathematical modelling^1^. A large body of literature has examined the effects of both contact structure and pathogen evolution on disease spread. On the one hand, recent models that account for realistic host population structure have shown that heterogeneity in host contact networks strongly influences the epidemiology of an infectious disease^2^,^3^,^4^,^5^. The presence of a few individuals with a disproportionately large number of contacts has been shown to significantly increase disease spread^3^,^4^,^5^, and these ‘superspreaders’ have been shown to be important drivers in many real disease outbreaks^6^. On the other hand, the constant and rapid evolution of pathogens in response to changing environments^7^,^8^ and medical interventions^9^ complicates disease control.

The intersection of these two fields, however, has received much less attention, although there are several reasons to think that contact structure can influence disease evolution. First, population structure can either amplify or suppress selection in simple population-genetic models^10^, but it is unclear to what extent these effects can be generalized to more complex infection dynamics. Second, models that have looked at the successive spread of two strains on a heterogeneous contact network have shown that the spread of the first strain modifies the network in a manner that may affect the spread of the second strain^11^,^12^,^13^,^14^. Third, local contact heterogeneity arising from spatial structure has been shown to affect the evolution of pathogen virulence both in theoretical^15^,^16^,^17^ and experimental investigations^18^. Given these findings, it is important to scrutinize in greater detail how contact structure influences the evolution of infectious diseases, and, moreover, whether there are particular contact networks that promote or hinder the invasion of new disease strains.

To this end, we use analytical and simulation methods to explore disease evolution in both empirical and idealized contact networks. We show that the heterogeneity in the host contact network that facilitates the spread of a single disease in turn lowers the fixation probability of an invading strain. Thus, many host contact structures may suppress the invasion of new disease strains and may slow disease evolution and adaptation.

## Results

### Simulation of disease evolution on networks

We gathered a set of empirically observed contact networks from different populations. Details of the networks, including sources, statistics and generative models, are given in Table 1 and the Methods. In brief, we use (i) a physical proximity network for students in a US elementary school; (ii) data from patients and health-care workers in a US hospital; (iii) a survey of the number of sexual contacts from the United Kingdom National Survey of Sexual Attitudes and Lifestyles (NATSAL); and (iv) a social network consisting of friends, family and co-workers from the US Framingham Heart Study (FHS). We contrasted these networks with a set of well-characterized theoretical networks including uniform random, Erdös-Rényí, scale-free and small-world. We characterize these networks using standard summary statistics from graph theory. An individual is said to have degree *k* if it is connected to *k* other individuals in the population, and the distribution of individuals’ degrees is given by the degree distribution, *p*(*k*).

**Figure 1:**
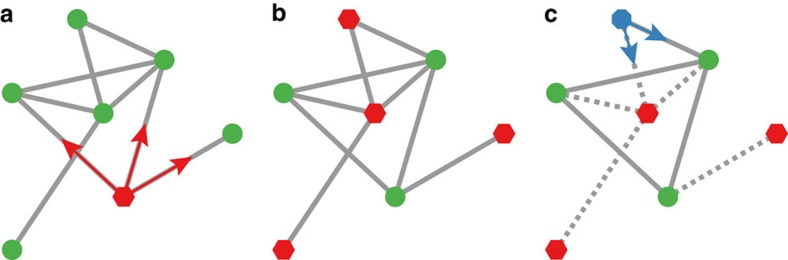
Model of disease evolution on networks. Individuals are represented by nodes in a network (shapes) and connections between individuals through which the disease can spread are represented by edges (grey lines). (**a**) In a population of initially susceptible individuals (green circles), a single individual becomes infected (red hexagons). (**b**) The infection spreads throughout the population, and eventually reaches a dynamic equilibrium (becomes ‘endemic’), where the number of new infections is balanced by the number of recoveries. (**c**) The pathogen in a single individual gains a beneficial mutation, creating a new pathogen strain (blue octagon). We are interested in the probability that this new strain fixes in the population, reaching endemic equilibrium and causing the resident strain to go extinct.

We simulated epidemics on these networks using a stochastic susceptible—infected—susceptible (SIS) model, which is a simplified representation of an endemic disease without lasting immunity^1^,^19^. We examine a series of steps in an epidemic caused by an evolving disease. In the first step (Fig. 1a), a single infected individual appears in the population due to, for example, migration from another population or infection from an external reservoir. Susceptible neighbours are infected with rate *β*_1_ and infected individuals recover with rate *γ*. The disease will either spread and reach an endemic equilibrium or go extinct. The probability that the disease does not immediately go extinct is the ‘emergence probability’, which is generally zero below threshold values of infectivity, *β*_1_, infectious period, 1/*γ*, and contact density. In the second step (Fig. 1b), the disease reaches an endemic equilibrium, where the prevalence remains approximately constant for long periods of time. The particular structure of the contact network is a strong determinant of prevalence patterns, such as number and degree distribution of infected individuals. In a third step (Fig. 1c), a second strain of the disease appears in a random infected individual. We assume that the mutation rate is sufficiently low such that the resident disease has time to reach an endemic level before a new mutant appears. The second strain infects susceptible neighbours with rate *β*_2_ and recovers with rate *γ*. We assume the competing strains induce perfect cross-immunity, such that there is no co-infection or super-infection. We are interested in the likelihood that a new strain takes over the population, causing the resident strain to go extinct. The second and third steps may repeat continually over the course of evolution.

### Selection exponents for empirical and theoretical networks

**Figure 2:**
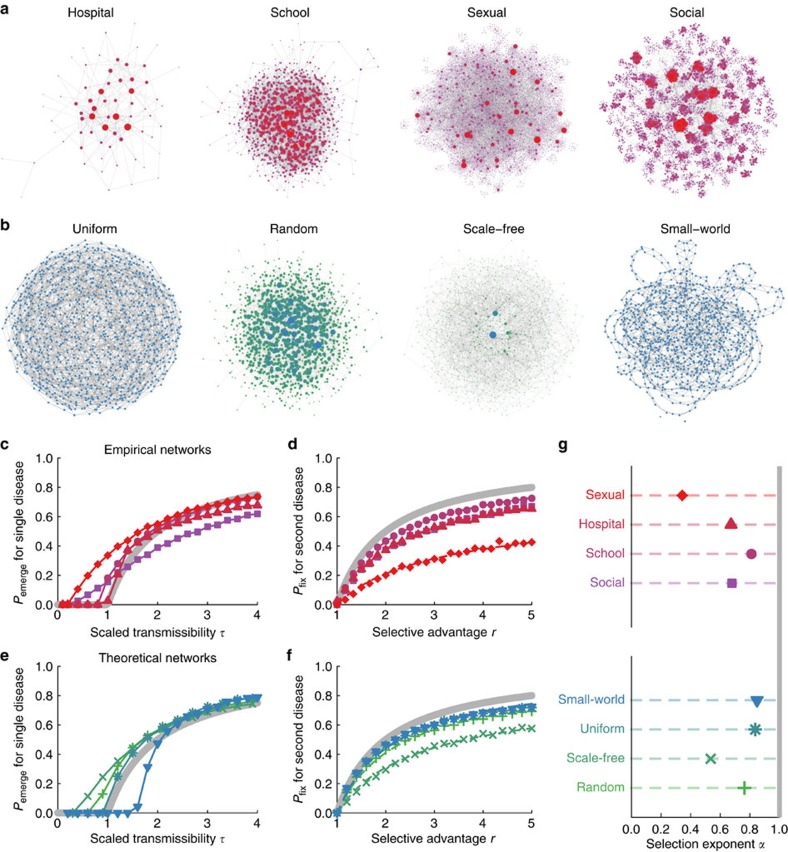
Network structure influences the evolution of diseases on real and theoretical contact networks. (**a**,**b**) Graphical representation of the networks. Large red or blue circles represent nodes with a high degree, small purple or green circles represent nodes with a low degree for the empirical and theoretical networks, respectively. (**c**,**e**) The probability that a single disease causes an epidemic (the emergence probability), *P*_emerge_, versus the scaled transmissibility *τ*=*β*(‹*k*›−1)/*γ*. *β* is varied and *τ* represents the expression for the basic reproductive ratio for the uniform network. The thick grey line indicates the emergence probability for a well-mixed network, *P*_emerge_=1−1/*R*_0_. The transmissibility value at which *P*_emerge_ becomes non-zero (that is, the epidemic threshold) depends on the network. (**d**,**f**) Dynamics of new disease variants. The probability of fixation, *P*_fix_, versus the selective advantage, *r*=*β*_2_/*β*_1_, of a new disease variant is strongly influenced by the population structure, but is not predicted by *P*_emerge_. The thick grey line indicates the fixation probability in a well-mixed network, 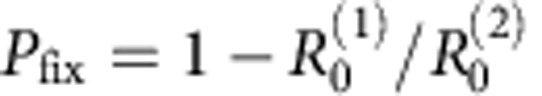. (**g**) The selection exponent, *α*, is calculated by fitting *P*_fix_ versus *r* to equation (27). Lower values mean that selection is suppressed compared to the uniform network. For a uniform or well-mixed population we expect *α*=1. Fits are shown by the solid lines in panels (**d**) and (**f**).

We found that the threshold infectivity required for the spread of a single strain differed strongly depending on the network structure (Fig. 2c,e). Heterogeneity in degree distribution facilitated disease spread, in agreement with previous work. The empirical networks generally had lower thresholds than the theoretical networks, apart from the scale-free network. We then analysed the ability of new strains to invade these networks (Fig. 2d,f). We define the fixation probability, *P*_fix_, as the proportion of simulations in which the new strain invades and drives the resident strain to extinction. Again, we observe large differences between networks, in this case in the dependence of fixation probability on the selective advantage, *r*=*β*_2_/*β*_1_. Contact structure lowered the fixation probabilities and thus inhibited the emergence of new disease strains. This finding is surprising given that all networks apart from the small-world facilitated the spread of an initial strain compared with a well-mixed population, by lowering the invasion threshold. Although this trend can also be observed in individual comparisons of two networks, it is not universal. For example, the uniform and small-world have different invasion thresholds but similar fixation probabilities, while the school and hospital networks have similar invasion thresholds but very different fixation probabilities. The fixation probability of the second strain can be written as a function of the selective advantage *r*, which is amplified or suppressed by a selection exponent *α*, such that *P*_fix_=1−1/*r*^*α*^ (see Methods and Lieberman *et al*.^10^). For uniform random networks, we expect *α*=1. Networks can thus be ranked based on their ability to promote selection of new strains by estimating a best-fit *α* from the curves in Fig. 2d,f. Interestingly, all the tested networks have an *α*<1 and thus suppress selection of beneficial mutants compared with the well-mixed or uniform case (Fig. 2g, Supplementary Table 1).

### Effect of degree heterogeneity on the fixation probability

Our goal is to understand the specific network properties that cause such disparate behaviours in disease evolution, but it is complicated by the fact that these example networks differ in many structural properties (Table 1). We thus examined classes of networks where single properties can be tuned.

**Figure 3:**
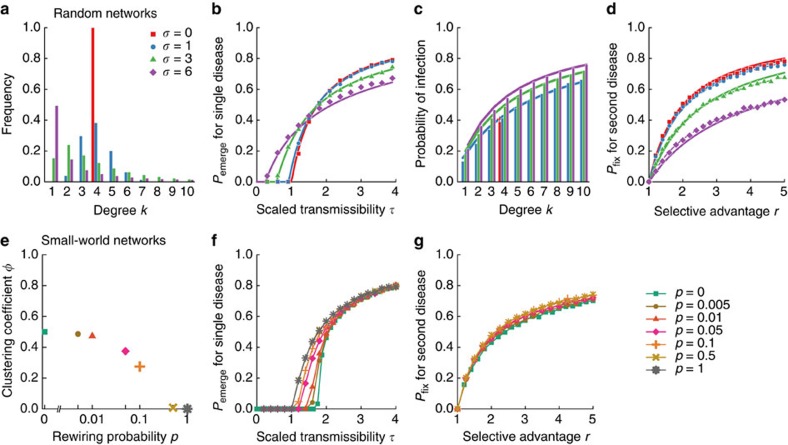
Dynamics of disease evolution in heterogeneous networks and small-world networks. (**a**–**d**) Heterogeneous networks; (**e**–**g**) small-world networks. (**a**) Degree distribution for upper panels is specified by a discrete gamma distribution (see Methods) with constant mean ‹*k*›=4 but tunable variance *σ*^2^. (**b**) The probability that a single disease causes an epidemic (the emergence probability), *P*_emerge_, versus the scaled transmissibility *τ*=*β*(‹*k*›−1)/*γ*. *β* is varied and *τ* represents the expression for the basic reproductive ratio for the uniform network. The multi-type branching process approximation (equation (9), solid lines) is in excellent agreement with the simulations (dots). (**c**) The probability of being infected at endemic equilibrium versus degree *k*. Predictions using pair-wise approximations (equation (17), lines) are in excellent agreement with simulations (bars). (**d**) The probability of fixation versus the selective advantage (*r*=*β*_2_/*β*_1_) of a new disease variant decreases for networks with larger variance in degree. Calculations from a new combined analytical technique (equation (18), solid lines) match well with simulations (dots). (**e**) For the lower panels, a set of small-world networks was created with constant homogeneous degree *k*=4 but varying clustering coefficient *φ*. (**f**) The probability of emergence for the first strain as a function of scaled transmissibility depends on clustering. (**g**) The fixation probability of the second strain as a function of the selective advantage, *r*=*β*_2_/*β*_1_, is independent of local clustering. For small-world networks, lines are simply connections between points to guide the eye.

Previous studies have identified individual variation in number of contacts as an important determinant of disease spread^1^,^3^,^20^. To examine the influence of degree heterogeneity on disease evolution, we constructed a series of networks with the same mean degree but tunable variance (Fig. 3a). We then compared various aspects of the simulation results with analytical approximations. In line with previous work, the threshold disease transmissibility required for emergence is lower for networks with higher variance in connectivity^3^,^20^. The emergence probabilities observed in the simulations (Fig. 3b) are well-approximated by a continuous-time multi-type branching process (detailed in Methods), where individuals are divided into types according to their degree. The probability of being infected at equilibrium as a function of the degree (Fig. 3c) is well described by a system of differential equations tracking pairs of individuals (see Methods and House and Keeling^21^).

Next, we examined the probability, *P*_fix_, that a novel strain, which appears at endemic equilibrium, displaces the resident strain. We find that this fixation probability depends highly on the network structure, and that it is markedly lower for networks with high variance in degree (Fig. 3d). Lower fixation probability results in slower adaptation when mutations are rare. Hence, heterogeneous contact structure acts to suppress selection for infectious diseases, despite facilitating initial spread.

We derive a combined analytical technique to approximate the invasion of a new disease strain into a population with a resident endemic disease, without the need for large-scale simulations (Methods). We first obtain the fraction of individuals with degree *k* who are susceptible at equilibrium, using a deterministic pair-wise approximation^21^. We then calculate the invasion probability of the second strain using a branching process approach. This calculation is similar to the single strain case, but incorporates the probability that an individual of degree *k* is susceptible at equilibrium. This combined pair-wise deterministic and multi-type branching process approximation is in excellent agreement with simulation results (Fig. 3d).

We also examined the effect of the degree of the focal individual in which the new strain arises on the fixation probability. The fixation probability is positively correlated with the degree of the focal individual and negatively correlated with the average degree of this individual’s neighbours.

### Effect of local clustering on the fixation probability

We next investigated the effect of local clustering on disease evolution. To this end, we constructed a series of small-world networks with fixed degree and tunable global clustering coefficient, *φ* (see Methods for definition and details). In brief, individuals in the network are initially connected to a local neighbourhood, after which a rewiring procedure is applied that introduces shortcuts in the network^22^. The emergence probability and endemic equilibrium for a single strain decrease with increasing clustering (Fig. 3e,f), in agreement with previous work^22^,^23^. Surprisingly, however, we find that the fixation probability of the second strain is completely independent of clustering (Fig. 3g).

**Figure 4:**
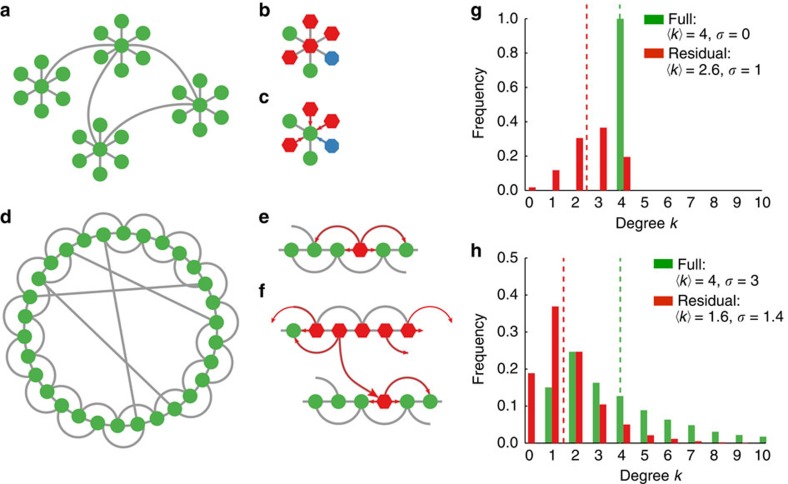
Mechanics of disease spread in theoretical networks. (**a**) Star graphs, or networks of interconnected stars, are an example of networks with large variance in degree distribution. Hubs facilitate the spread of the first disease in a susceptible population. (**b**) New disease strains are likely to appear in the leaves. Hubs are likely to be already infected, hindering invasion. (**c**) Susceptible hubs are likely to be quickly reinfected by leaves infected with the resident strain. Therefore, the fixation probability of new strains on star-like graphs is low. (**d**) Small-world networks are made up of mainly local connections with variable rewiring to create shortcuts. (**e**) The initially infected individual can potentially infect all its neighbours, while those subsequently infected have more limited options. (**f**) Shortcuts allow the disease to jump to fully susceptible areas of the network, facilitating spread. They are less important for the second disease, as all parts of the network are already infected. Therefore, fixation probability of new strains on uniform, locally connected networks does not depend on the rewiring probability. (**g**,**h**) The degree distribution of the full network (green; left) is compared with the residual network (red; right) of susceptible individuals remaining at a particular time point during endemic equilibrium. (**g**) Uniform network (equivalent to gamma-distributed network with *σ*=0). (**h**) Gamma network with *σ*=3. Dotted lines represent the mean of the distributions.

Our results suggest that degree heterogeneity more strongly influences disease evolution than local clustering. The intuition behind these results is illustrated in Fig. 4. For a first strain spreading in a fully susceptible population, high variance in degree facilitates spread due to the the presence of easily accessible hubs with high connectivity. These hubs, once infected, markedly reduce the extinction probability as they are surrounded by many susceptible individuals. Star-like graphs are an extreme example of this situation (Fig. 4a). When a novel mutant arises in endemically infected populations, however, the hub-individuals are the most likely to already be infected by the resident strain, and hence are unlikely to be available for infection by the mutant. The higher the variance, the stronger this hub-holding effect, and the lower the average degree of remaining susceptible individuals. As there are many more peripheral than centre nodes in the star graph, a randomly introduced new strain is more likely to appear in a peripheral node (Fig. 4b). To spread, however, the new strain must first infect a centre node, which is very likely already infected with the resident strain. If the centre node recovers before the mutant goes extinct, the mutant has the possibility to infect it; however, it must do so before the centre is reinfected by the resident strain from one of the many other infected peripheral nodes (Fig. 4c).

To understand the lack of influence of local clustering, we consider a small-world network (Fig. 4d–f). When the first strain spreads in a fully susceptible population, patient zero has the possibility to infect all of its neighbours. Strong local clustering implies strong overlap in neighbours of two connected individuals. As the epidemic progresses, the neighbours of subsequently infected nodes are likely to already be infected. Consequently, small-world networks with large clustering coefficients tend to decrease the probability that a disease will emerge. In fully susceptible populations, shortcuts facilitate disease spread by allowing the strain to jump to new areas of the network where most individuals are still susceptible. These shortcuts do not help the spread of a second strain, as jumping to a different part of the network is not beneficial when the resident disease is endemic throughout the network. Hence, the fixation probability of new strains is independent of the rewiring probability.

This intuitive argumentation can also be formulated in terms of the effect the first strain has on the degree distribution of susceptible individuals. As individuals with high degree are more likely to be infected at equilibrium (Fig. 3c), the mean degree in the residual network of susceptibles^5^,^24^ decreases with increasing variance (Fig. 4g,h). For uniform networks, the variance in degrees can only increase between initial and residual networks, while for more heterogeneous networks the residual network could have lower variance. Note that, in the SIS model, as recovery and reinfection are constantly occurring, the residual network is a dynamic concept: the actual nodes in it may change while the average properties remain constant.

## Discussion

Throughout this paper, we have considered one particular two-step model of competition between different strains of an infectious disease spreading on a static contact network. A single pathogen strain obeying SIS dynamics spreads in a host population until it reaches endemic equilibrium. The probability of successful spread increases with increasing degree heterogeneity of the host population. In the endemic equilibrium, a new strain with complete cross-immunity, differing only in its transmission rate, appears in a single infected individual. Conversely to the initial spread, the probability that this new strain can successfully invade the host population decreases with increasing degree heterogeneity. Such a model is a good description of an endemic disease where the transmission of *de-novo* mutants is a rare event. To understand in what way the results may be generalizable to other models of disease spread, it is important to discuss some of implications of the model assumptions.

The SIS model used in this paper is the simplest mathematical model of an endemic disease^1^. Endemic diseases are at an increased risk of continual evolution when compared with single-wave epidemics, with the latter being better described by variations of the susceptible—infected—removed (SIR) models. The type of analysis presented here is not appropriate for such single-wave outbreaks, due to the absence of an endemic state. The effect of spatial structure on two subsequent epidemic waves of new strains in SIR-type models has been considered elsewhere^11^,^12^,^14^. For an endemic disease with temporary immunity that can be described by a susceptible—infected—removed—susceptible (SIRS) model, we hypothesize that the same general trends we see for the SIS apply. Temporarily recovered individuals are not available to reinfection by either strain. From the point-of-view of the invading mutant strain, the hub-holding effect of infected individuals also applies to temporarily recovered individuals, thus hindering fixation in populations with high variance in degree.

In this study we considered beneficial mutant strains with increased transmissibility, *β*. Alternatively, a longer infectious period (smaller γ) would also convey a benefit in well-mixed populations^16^. We repeated our analysis for a second strain with a smaller recovery rate, and found that the general trends with regards to degree heterogeneity were identical, as expected from the analytical results (Supplementary Fig. 1). However, we also observed that the fixation probability and selection coefficient were consistently higher when *γ*_2_ was varied as opposed to *β*_2_, keeping the ratio 
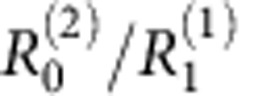
 constant. This effect is likely related to our previous results that, in small, well-mixed populations, when both transmissibility and recovery can vary independently, the direction of selection is shifted towards decreasing the recovery rate, as opposed to simply increasing *R*_0_ (ref. 25). Network structure may skew selection in a similar way as small populations. Further complications arise in models for the evolution of virulence, where *β* and *γ* are not independent. Previous work on the evolution of virulence has shown that the evolutionarily optimal virulence level is different in structure populations as compared with well-mixed populations^15^,^16^,^17^,^26^,^27^. This work, however, has not investigated the specific network properties that modulate these trends. Future work will be needed to fully understand all the factors influencing disease evolution in more realistic scenarios.

We chose to model the introduction of a new disease strain by randomly choosing a single individual who was infected with the first strain during the endemic phase, and instantaneously switching their status to infected with the second disease strain. With this procedure, we aim to simulate the situation where within-host evolution of a pathogen leads to a novel strain emerging. Alternatively, new disease strains could be introduced into a population from an external reservoir or another disconnected population. In this case, it may be more realistic to consider introduction into a random susceptible individual, or any random individual. As susceptible and infected individuals are spatially clustered within a contact network at endemic equilibrium^23^, changing these initial conditions can affect the emergence probability. We verified that our results are qualitatively identical with this alternate initial condition, and can similarly be well-approximated analytically, with the appropriate updates to equations (18) and (23).

In our simulations we consider perfect cross-immunity or competitive exclusion within a host. That is, being infected with one strain protects against infection by the other strain. Alternatively, hosts can be simultaneously infected with multiple strains (co-infection) or the strain currently infecting a particular host can be displaced by infection with another strain (super-infection). The effect of imperfect cross-immunity will strongly depend on the way in which the two strains influence each other’s infection and recovery rates, and will vary depending on the particular real-world disease considered. Perfect cross-immunity implies that infection with one strain completely blocks infections by the other strain. Conversely, complete lack of cross-immunity implies that the two strains do not influence each other’s infection rates. In this case, the invasion dynamics of the second strain will be equivalent to the case where the first strain is absent. The effect of contact structure on the invasion of the second strain with partial cross-immunity will thus be in between these two extremes.

In this paper, we have modelled population structure as a static, unweighted network. Contacts between individuals in many real-world situations, however, are dynamic. For example, infected individuals may stay at home or may be quarantined when infected, or move from home and the workplace to a hospital. If contacts between individuals are updated in a manner that is independent of disease status, we expect to find qualitatively similar results as in the static case. Such rewiring may dampen the effect of population structure as it either changes the instantaneous degree distribution of the network or maintains the degree distribution but re-assorts neighbours. The effect of such rewiring will also depend on the timescales at which contacts are updated in comparison with the timescale of disease spread. Previous work by Cross *et al*.^28^ demonstrated that, for a metapopulation with intergroup migration, the spread of a single disease depended critically on the relative timescales of recovery and migration, and it is likely that disease evolution in this context may be similarly influenced by these parameters.

By considering competition between at most two strains, and requiring that the second strain arise only after the first is at a quasi-steady-state, we have implicitly assumed that there is a separation of timescales between the epidemic and evolutionary processes. In our analysis, we focused only on the ultimate probability of fixation, and not the time required to reach fixation, which may be very long for certain population structures (those with more local and less global connections and thus higher clustering coefficients), and when strains are close in fitness. If we relaxed the separation of timescales assumption and instead allowed mutations to occur at a constant rate in each infected individual, we may observe situations where multiple strains coexist for very long periods of time. Other work has focused on the role of population structure in facilitating pathogen diversity in this regime^29^,^30^.

In conclusion, we show that heterogeneity in contact structure suppresses disease evolution by lowering the fixation probability of any newly arising disease strains. This finding is surprising in two ways. First, the suppressive effect on evolution is in contrast to the well-established finding that contact heterogeneity otherwise facilitates the initial spread of a disease^2^,^3^,^4^,^5^. However, the effect makes sense in light of the earlier finding that the initial strain modifies the residual network of susceptibles^11^,^12^,^13^,^14^. Second, the suppressive effect is also in contrast to the earlier finding that certain network structures can amplify selection^10^. This discrepancy arises from the differences in the underlying population dynamic model used to consider competition between two genotypes. Previous work used the Moran process model of reproduction and death, which considers only two types of individuals, while we use an infectious disease model that requires tracking susceptibles along with two types of infecteds. Our results highlight the fact that findings from the Moran model, such as the universality of fixation probabilities in isothermal graphs^10^,^31^, may have little bearing on infectious disease dynamics. Despite the inherent challenges, understanding the interaction between disease emergence, evolution and contact structure is highly relevant to infectious disease epidemiology, as continual evolution is a major barrier to control, and interventions that target contact structure are increasingly popular.

## Methods

### Simulation details

All simulations were implemented as a Gillespie next-reaction method. For single-disease simulations, the infection is introduced into one random individual, and the simulation is run until the disease is extinct or reaches a quasi-steady state (QSS), or *t*_max_=1,000, whichever occurs first. QSS is defined when there is <2% difference between the average prevalence over the last third of the total simulation time and the middle third, after an initial burn in period of *t*_burn_=100/*γ*. For the small-world network with no rewiring, it was necessary to increase *t*_max_ to 10,000 and *t*_burn_ to 1,000/*γ*. The emergence probability is calculated as the fraction of runs out of where the disease does not go extinct. At least 7,000 runs were simulated for each parameter value.

For the two-strain invasion simulations, the resident strain is first introduced at a high level to avoid early extinction and allowed to reach a QSS (waiting at least *t*_burn_). Then a single-infected individual is randomly chosen to be infected with the mutant strain. The fixation probability is calculated as the mean fraction of invasion attempts where the resident strain goes extinct while the invading strain still remains. Runs where both disease strains remained after *t*_max_ were rare and not included in the reported results. New networks were randomly generated for each simulation run, resulting in at least 6,000 invasion attempts per parameter.

The value of *β*_1_ at which the mutant strain is introduced was chosen so that the QSS level was approximately equal for all networks. For Fig. 2d,f (empirical and theoretical networks) and Fig. 3g (small-world networks), we used *β*_1_(‹*k*›−1)/*γ*_1_=3, and for Figs 3d and 4 h (gamma-distributed networks), we used *β*_1_(‹*k*›−1)/*γ*_1_=1.5. Changing these values did not change the trends observed unless the QSS level was very different between networks.

### Clustering coefficient

The clustering coefficient, *φ*, is also known as ‘global clustering coefficient’ or ‘transitivity’^2^,^23^. It is defined as the ratio of the number of triangles in the network (sets of three nodes each connected to each other) to the number of triplets (set of three nodes with at least two connections between them). If *A* is the adjacency matrix of the network, then


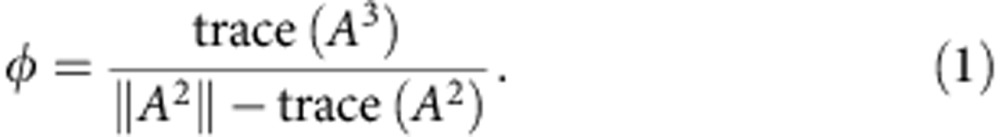


### Network generation algorithms

For the uniform network, all individuals have the same degree. We use the configuration model, expressed with a stub-connection algorithm, to create random graphs with a specified degree distribution. By randomly connecting individuals we reduce higher-order structure^32^. For each node we first assign it a degree *k*, and then create a set of *k* stubs that represent each of these edges with only a single tail connected to a node. We repeat this for all nodes and then combine these stubs into a master set. This set is then randomly divided in half, and a stub from each subset is matched to one from the other subset, forming a complete edge. If there is an uneven number of stubs, a random individual is given an extra stub. We do not allow self connections or duplicate edges between nodes.

For the gamma-distributed-degree network, each individual is assigned a degree drawn from a discretized version of the gamma distribution with a mean degree ‹*k*› and s.d. *σ*_*k*_. The gamma distribution was chosen because it allows the mean and variance to be varied independently, with any variance between zero and infinity possible. Discretization was performed by first drawing a random number from a continuous gamma distribution with mean ‹*k*›−1 and s.d. *σ*_*k*_, rounding to the nearest integer, and then adding 1. It was confirmed numerically that this created a distribution with the desired properties over the required range of ‹*k*› and *σ*_*k*_ values. The network is then created using the stub-connect algorithm (see above).

For the random network, we use the Erdös-Rényí/Gilbert model^33^,^34^. An edge is constructed between each pair of individuals with a probability *p*, independent of the existence of other edges. The resulting degree distribution is binomial, with mean degree ‹*k*›=*p*(*N*−1).

For the small-world network, we use the method described by Santos *et al*.^22^ Each individual is first arranged in a ring, and then connected to its *m*=‹*k*›/2 nearest neighbours on either side. Each edge of every node is then rewired with probability *p*. Rewiring involves disconnecting from the distal node and connecting to another random non-self and non-neighbour node, such that dual edges are avoided and the uniform degree of the network is preserved.

For the scale-free network, we use the Barabási-Albert model of preferential attachment^35^. The network starts as a fully connected group of *m*=‹*k*›/2 nodes. Each new node is added to the network and connected to *m* other individuals, each with a probability proportional to the individuals current degree. This creates a network with a degree distribution following a power law, *p*(*k*)∝*k*^−*v*^, with the exponent *v*=3 and average degree ‹*k*›.

### Empirical networks

For the FHS—social contact network, we used a previously described network of social contacts that was collected as a part of the Framingham Heart Study^36^,^37^. Individuals participating in the study were connected to family members, co-workers and self-reported friends who were also enrolled in the study. This network was available for seven examinations between 1971 and 2000, and we chose the earliest time point, when the network was the largest. This network represented 5,253 individuals who were connected to at least one other individual. The average degree was ‹*k*›=6.5, the s.d. in degree was *σ*_*k*_=6.8, and the clustering coefficient was *φ*=0.68.

For the school contact network, we worked with a publicly available contact network observed among students and teachers at an elementary school over a single school day^38^. Participants wore electronic sensors that detected close physical proximity and recorded the times over which these contacts occurred. This network is therefore either dynamic (if we consider an edge existing between individuals at time *t* provided they are in close proximity at that point) or weighted (if we sum up the total time two individuals spent within close proximity over the whole day). To simplify analysis and facilitate comparison with other example networks, we sampled a static, unweighted subnetwork from the full network by connecting every individual with a probability proportional to the total contact time. From this sampled network, we chose the giant component to ensure the population was a single connected graph. With this method the average degree is a free parameter determined by scaling the probability of each edge, and we chose it to agree with the FHS social network. The result was a network with 740 individuals, with an average degree ‹*k*›=6.5, s.d. in degree *σ*_*k*_=3.3, and clustering coefficient *φ*=0.04.

For the hospital contact network, we used data collected in a hospital setting to create a contact network of health-care workers and patients, which is freely available online at http://www.sociopatterns.org^39^. Similarly to the school network, participants wore electronic sensors and incidences of close physical proximity were recorded over 5 days, resulting in a dynamic/weighted network. We again sampled this network to generate a static, unweighted network with a single giant component. The result was a network with 68 individuals, with an average degree ‹*k*›=6.5, s.d. in degree *σ*_*k*_=5.3, and clustering coefficient *φ*=0.29.

For the NATSAL—sexual contact network, we used the results from the United Kingdom National Survey of Sexual Attitudes and Lifestyles (NATSAL) that is freely available online at http://www.natsal.ac.uk/ and has been published previously^40^. This survey collected the number of sexual partners over the last 5 years for a population of around 30,000 individuals (combining the NATSAL-1 and NATSAL-2 studies in 1990 and 2000). This degree distribution fits very well to a power law function^41^, 
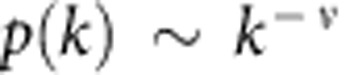
, with exponent *ν*=2.5, *k*_min_=1, and 
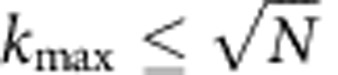
. We then generated a degree sequence for *N*=10,000 nodes and maximum degree 
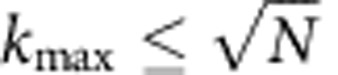
 that follows a power law with exponent *ν*, created a network from this degree sequence using the stub-connect algorithm described above for random networks. We extracted the giant component, resulting in a random network with an average 7,578 individuals, mean degree ‹*k*›=2.7, mean s.d. in degree *σ*_*k*_=4.9 and clustering coefficient *φ*=0.002.

### Branching process calculations for disease emergence in networks

In the early stages of infection, when the number of infected individuals is very low, the SIS model in a heterogeneous host population can be approximated by a multi-type branching process. The particular stochastic process we choose to describe the epidemic is related to the ‘continuous offspring production’ model discussed in the viral dynamics literature^42^ and has been previously used to study disease emergence^43^. Each individual of type *i* has a constant rate, *r*_*ij*_, of producing infected individuals of type *j* and also a constant rate of recovery, *γ*, akin to death with immediate replacement in other models. Other stochastic processes used to describe the initial phase of epidemics include ‘burst models’ where offspring distributions are specified *a priori* (such as Kronecker delta^42^ or Poisson^44^,^45^), percolation models^46^, or independent infection probabilities^47^. The continuous offspring production model considered here mimics what occurs in most simulation algorithms, including ours, and may more closely represent biological reality. The offspring distribution is calculated *a posteriori* to be multinomial.

An important quantity to calculate is the probability generating function (PGF), *F*_*i*_(***s***), for the number of secondary infections of individuals of each type, ***s***=(*s*_*i*_, *s*_2_, …, *s*_*n*_), caused by a single-infected individual of type *i*. For this process, we derive


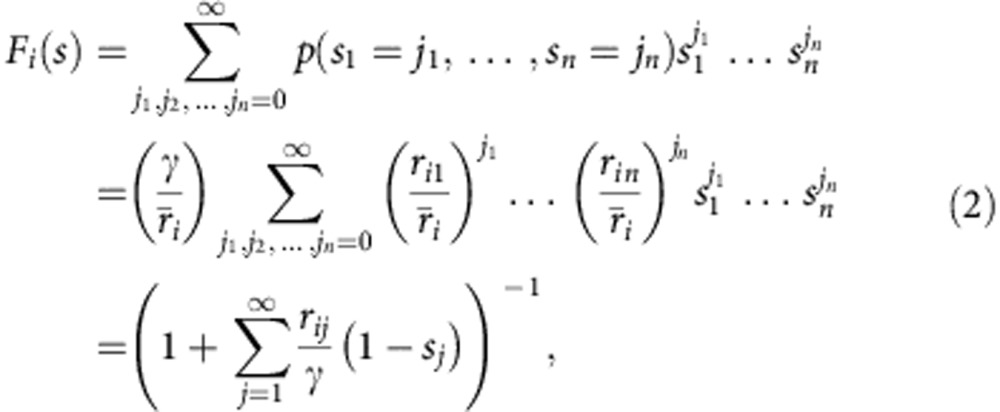


where *j* and (*j*_1_,*j*_2_,…,*j*_*n*_) are indices, *n* is the total number of different types of individuals, and 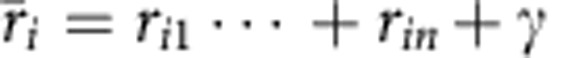 is the sum of all rates.

Using the definition of the basic reproductive ratio, *R*_0_, as the average number of secondary infections produced by a single-infected individual, we can define multi-type reproductive ratios^44^,^45^,


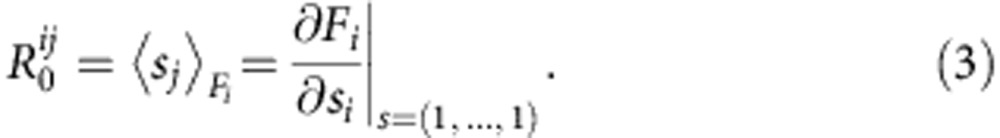


This allows us to use 
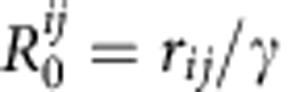
 to write the PGF as


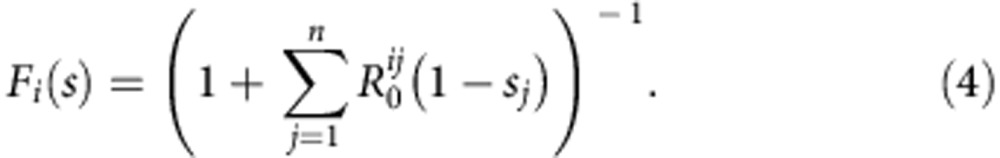


We now consider a network-structured population, where individuals are classified according to their degree *k*. Individuals of type *i* are those who are connected to exactly *k*_*i*_ other individuals. The frequency of individuals of degree *i* is given by *p*(*i*). Following Yates *et al*.^45^, we can break down 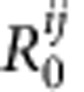 in terms of the disease factors and structural factors,


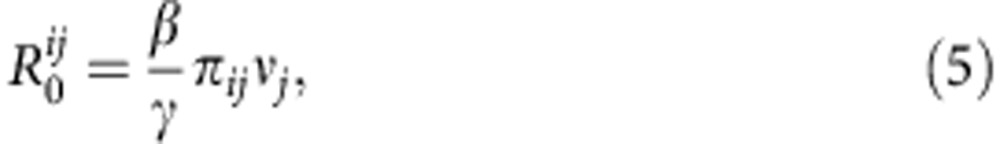


where *β* is the per-contact transmissibility of the disease, *π*_*ij*_ is the average number of type *j* contacts a type *i* individual has (the mixing matrix), and *ν*_*j*_ is the susceptibility of type *j* individual (1=fully susceptible, 0=fully resistant to infection). If we assume the network is constructed by the configuration model, that is, edges are joined randomly and there is no correlation between the degree of individuals on either side of an edge, then,


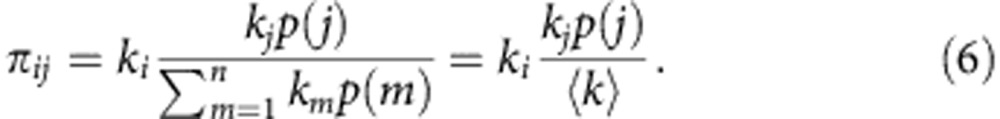


As we are interested in a fixed network structure, we immediately encounter a problem that does not occur when considering heterogeneous yet mixing populations. For all individuals other than the very first infected, the actual number of susceptible contacts will be one less than that given by *π*_*ij*_, because the contact from whom the infection originated cannot be reinfected. For these secondary infections, we must consider the modified mixing matrix,


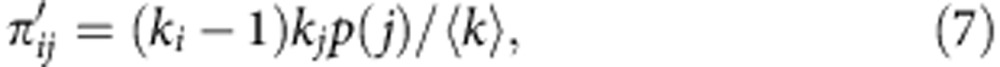


based on the concept of ‘excess degree’^48^, and hence a modified reproductive ratio 
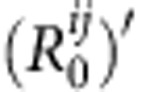
.

We want to calculate the probability that a disease introduced into a population causes an epidemic, as opposed to going extinct. In a random mixing population, standard branching process theory gives the ultimate extinction probability, for an infection originating in a type *i* individual, as the solution to *x*_*i*_=*F*_*i*_(***x***). Taking into account the difference between those infected in first and later generation, we must first find the extinction probability for all those infected in later generation and then calculate the ultimate extinction probability starting from a single infection^47^,^49^,


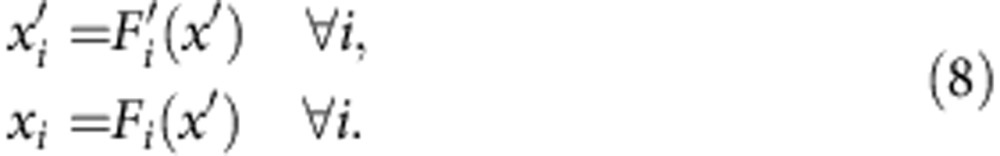


The emergence probability is then given by


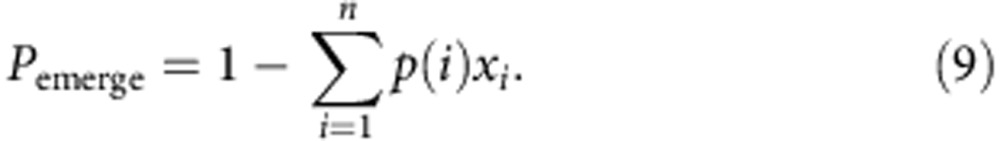


For a homogeneous, well-mixed population, this calculation reduces to


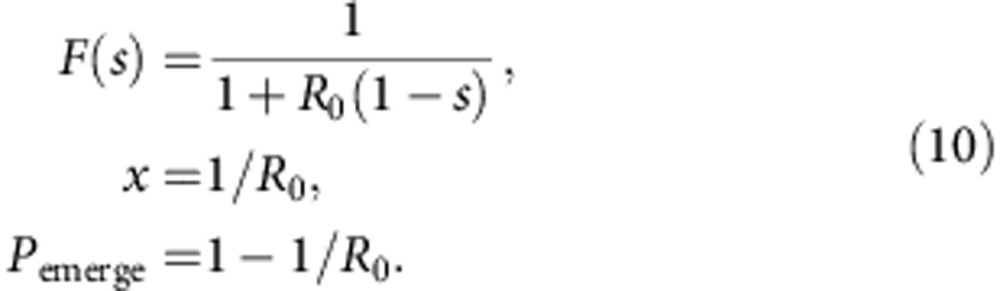


For a homogeneous population with a fixed network structure with degree *k* we only have a single type *k*, such that *π*_*ij*_≡*π*=*k* and 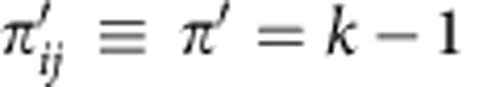, and the calculation reduces to


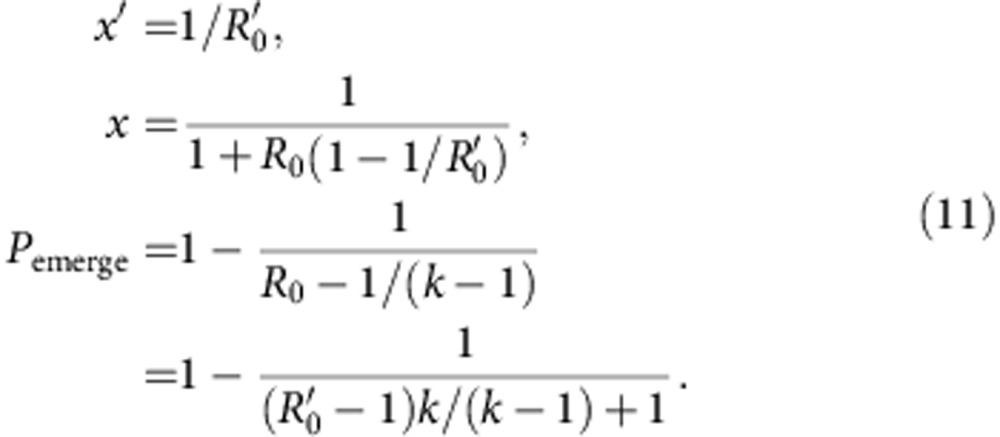


We can see from the first expression for *P*_emerge_ above that, for a homogeneous fixed network structure, *P*_emerge_≤1−1/*R*_0_ and, that *P*_emerge_=0 when 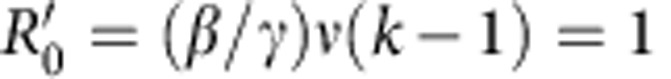.

There are certain limitations to this technique for estimating the emergence probability of diseases in networks. First, we assume an infinitely large random network. Second, host heterogeneity is modelled by dividing individuals into groups based on their degree. Hence, higher order structure is ignored, and so networks that contain high levels of assortativity or clustering may not be well represented with this method. The presence of clustering will decrease *P*_emerge_, while assortativity could either increase or decrease it. This method also ignores the issue that, in the SIS model (as opposed to the often considered SIR model), a recovered individual could become reinfected during early emergence, increasing *P*_emerge_. Hence, the branching process might underestimate the true probability of emergence for the SIS model, even in a well-mixed population.

### Pair-wise equations for equilibrium disease behaviour in networks

Branching process calculations can tell us about the probability of disease emergence by capturing stochastic effects that are important when disease levels are low, but do not accurately capture the dynamics as prevalence levels become significant. For this task, deterministic models that track both infected and susceptible individuals are appropriate.

We use the method of pair-wise equations^23^ to describe disease dynamics in a network-structured population. We start with the full SIS pair-wise equations^50^,


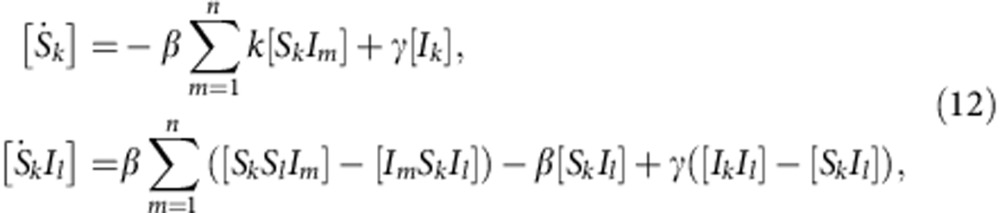


where [*A*_*k*_] describes the number of individuals with degree *k* that are in state *A*, [*A*_*k*_*B*_*m*_] describes the number of pairs of individuals where one member of the pair has degree *k* and is in state *A* and the other member is in state *B* with degree *m*, and [*A*_*k*_*B*_*m*_*C*_*l*_] is analogous but for triples, with *B* being the middle member. As the total size and structure of the population is constant, we can use [*I*_*k*_]=[*k*]−[*S*_*k*_], where [*k*] is the total number of individuals with degree *k*, [*k*]=*p*(*k*)*N*. These equations are exact, but, to completely describe the system, equations for higher order groups of individuals are needed, making them intractable. We make the following series of common approximations (detailed in House and Keeling^21^) to close the equations:

(i) triple closure,


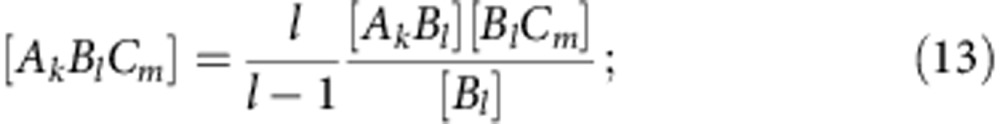


(ii) deconvolution of pairs,


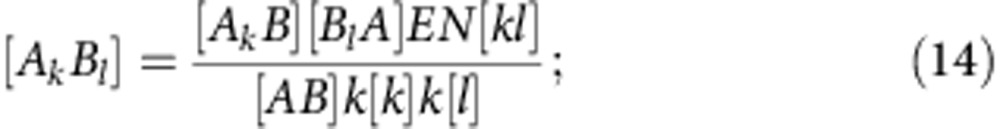


(iii) detailed balance,


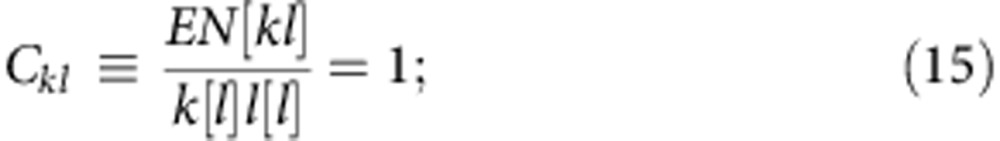


(iv) deconvolution of individuals,


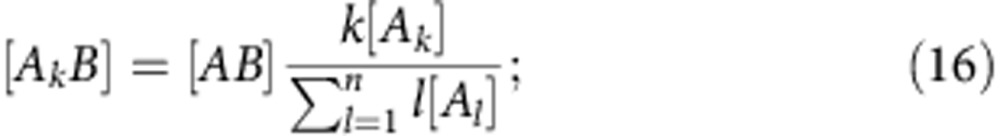


where *N* is the total population size and *E* is the total number of edges. Whenever a disease state occurs without a subscript, it implies that it includes the sum over all degrees. We then arrive at a simplified set of equations,


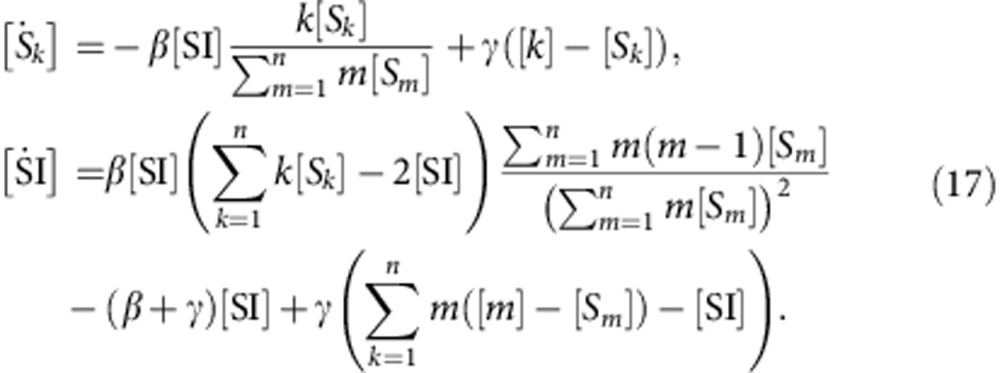


[SI] is the number of edges between susceptible and infected individuals. These equations can be used to describe the time course of the infection among individuals of each degree and the equilibrium state.

Deriving a closed and reduced set of pair-wise equations requires making approximations about the types of higher-order correlations between connected individuals introduced by the epidemic. Triple closure and deconvolution of pairs and individuals are examples of such approximations. It is difficult to formulate exactly when these assumptions hold *a priori*, but previous studies have shown that they usually agree very well with simulations^21^. In contrast, the detailed balance approximation depends only on the network structure and is assured in a configuration model. In networks with other methods of edge creation, such as preferential attachment, this simplification may fail. As stated above, these approximations assume that the network clustering, *φ*, is zero, although corrections can be made to account for non-zero values^21^.

### Combining techniques to approximate invasion of a second disease

We derive a combined analytic technique to approximate the invasion of a second disease {*β*_2_,*γ*_2_} in a population infected with a resident disease {*β*_1_,*γ*_1_} at endemic equilibrium. We first solve for the steady state of the pair-wise equations for {*β*_1_,*γ*_1_} (equation (17)), which can give us both the total fraction infected with the first disease *f*_*I*_=1−∑ [*S*_*k*_]/*N* and the fraction of degree *k* remaining susceptible *ν*_*k*_=[*S*_*k*_]/(*Np*(*k*)). We then used this *ν* along with {*β*_2_,*γ*_2_} to determine the effective basic reproductive ratios for the second disease, 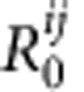 and 
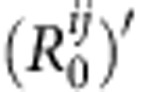
, which can then be used in the branching process calculation to determine the emergence probability (equations (4)–(9), 12, 13, 14, 16, 17). Using this procedure, the resulting emergence probability, *P*_emerge_, is equivalent to the fixation probability, *P*_fix_, for the second disease. However, to account for the fact that, in our simulations, we only allow the second disease to arise in an individual who was already infected with the first disease, we modified to equation (9),


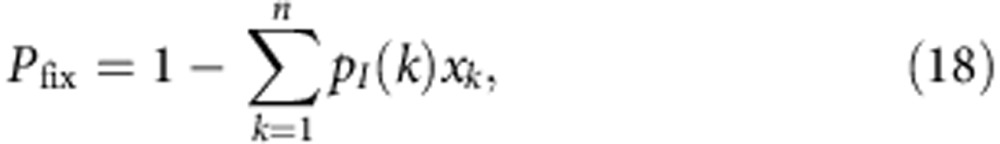


where *p*_*I*_(*k*)=[*I*_*k*_]/[*I*]=*p*(*k*)(1−*ν*_*k*_)/*f*_*I*_ is the probability that a randomly chosen infected individual has degree *k*.

Another method of combining deterministic and stochastic approaches was recently derived independently to study disease adaptation during emergence in well-mixed populations^51^.

We found that this first-order approach consistently underestimated the fixation probability of the invading disease, which we hypothesized was due to assuming an individual’s susceptibility, *ν*_*j*_, was independent of the fact that the connected individual who might infect them was also susceptible. To derive a second-order approximation, we took the number of susceptible neighbours directly from the pair-wise equations and used equations (14)–(16), 25, 26 to arrive at


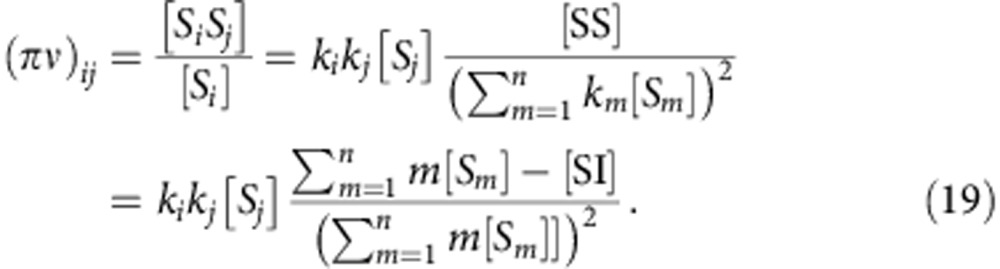


The terms [SI] and [*S*_*j*_] can be determined numerically from the equilibrium of equation (17), and by definition 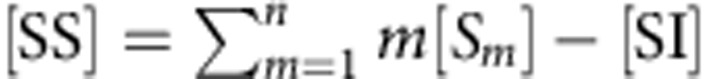.

Recall that in the one disease case, the mixing matrix *π*_*ij*_ was,


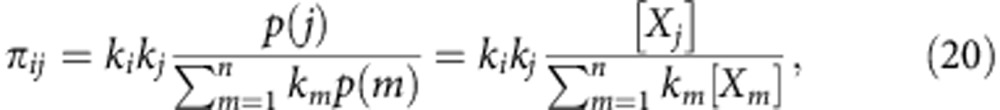


where *X*_*j*_ signifies an individual of type *j* in either state *S* or *I*. We can then write the new mixing matrix 
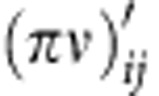
 in terms of *π*_*ij*_,


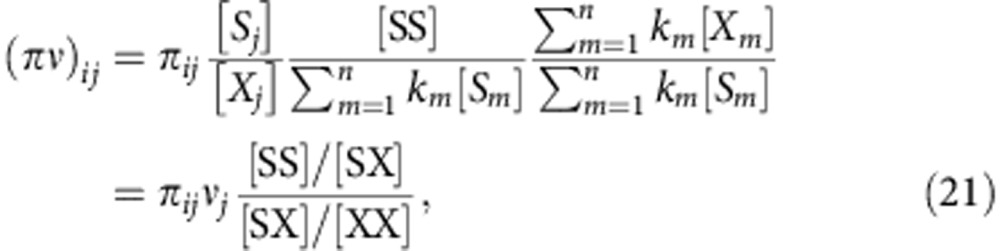


where *ν*_*j*_=[*S*_*j*_]/[*X*_*j*_] is the fraction of susceptible individuals of type *j*, 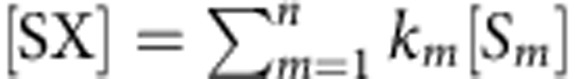 is the number of edges from a susceptible individual to any individual and 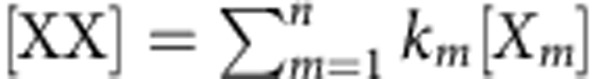 is the total number of edges. From the last line we can see that (*πν*)_*ij*_ modifies *π*_*ij*_*ν*_*j*_ by taking into account the fact that, because of clustering of susceptible individuals, the fraction of a given susceptible individual’s contacts that are still susceptible (top fraction) may be higher than the no-clustering expectation, and therefore may more than compensate for the fact that the individuals who remain susceptible have a lower number of contacts on average (bottom fraction).

This value of (*πν*)_*ij*_ cannot be used directly. For secondary infections, we must again consider a modified value 
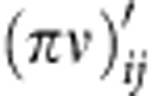
, where *k*_*i*_ is replaced by *k*_*i*_−1 in equation (19) to take into account the fact that the neighbour from whom the infection originated cannot be reinfected. The resulting expression is


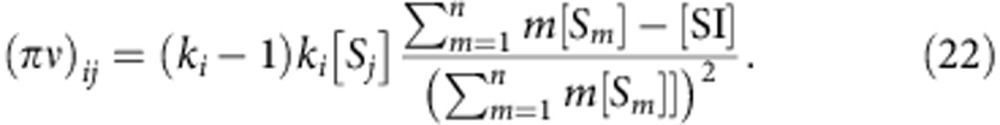


We also need to take into account that the individual who is first infected with the second strain was already infected with the first disease, and so for primary infections the mixing term becomes


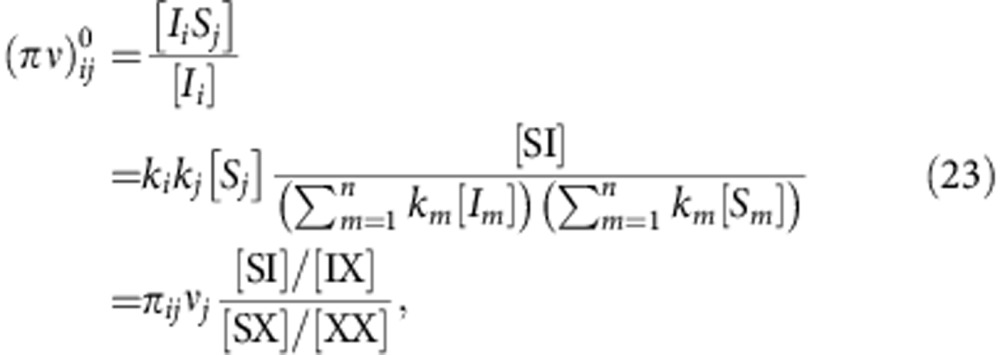


where 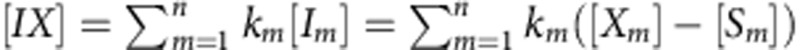 is the number of edges from an infected individual to any individual.

Therefore, to produce the analytical approximations for the fixation probability of an invading strain displayed in Fig. 3d, we first derived 
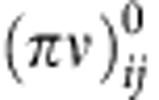
 and 
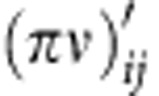
 from equations (19) and (23), using the solutions to the pair-wise equations for the first disease (equation (17) with {*β*_1_,*γ*_1_}). We can then derive 
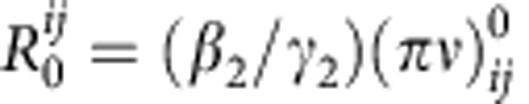
 and 
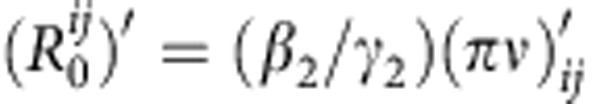
, respectively. These reproductive ratios were then substituted into equations (4) and (8) to obtain ***x***, which was then used in equation (18) to determine the fixation probability of the second disease.

### Analytical solution for the uniform network

The quasi-steady-state distribution of individuals infected with the first at endemic equilibrium can be estimated using the pair-wise equation (17). For a homogeneous population with a fixed random network structure with degree *k*, [*S*_*k*_]≡[*S*], and these equations reduce to


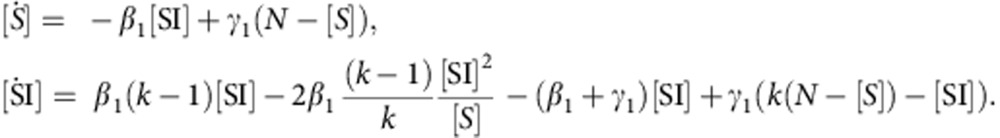


The non-zero equilibrium states for this system, [*S*]^∞^ and [SI]^∞^, are


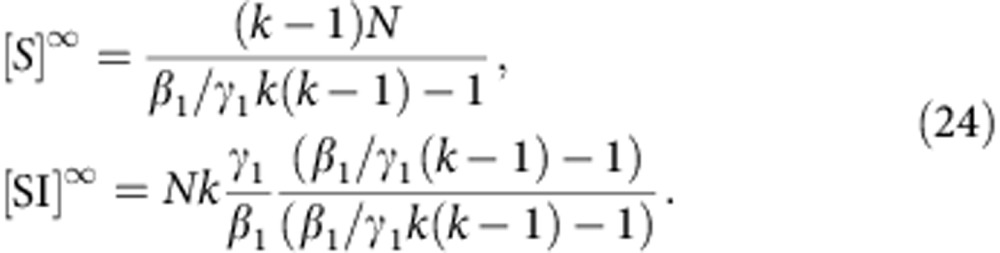


To calculate the fixation probability of the second disease, we need to derive (*πν*)^0^ and (*πν*)′ for the uniform network. Substituting the equilibrium conditions into equations (23) and (22), we arrive at


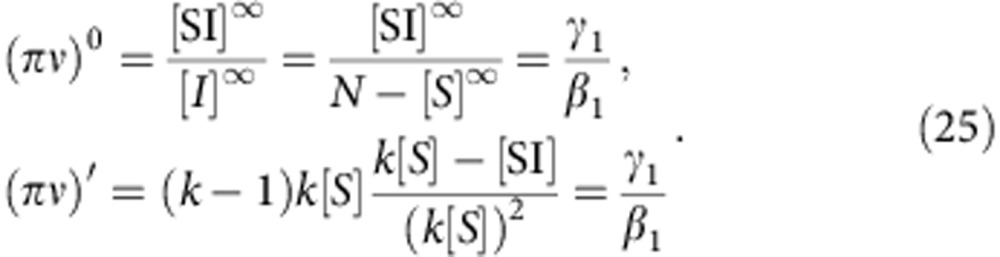


Finally, the fixation probability can be found by solving equations (8) and (9) with *R*_0_=(*β*_2_/*γ*_2_)(*πν*)^0^ and 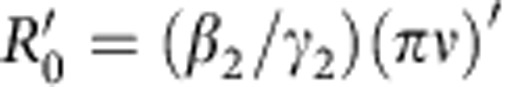,


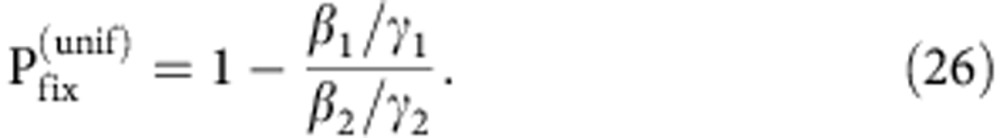


### Deriving the selection exponent

For non-uniform networks, there is no general analytic expression that allows us to directly quantify the relationship between network heterogeneity and fixation probability for an invading strain. While the method can be implemented numerically, we chose to also use an empirical function to model the trends observed in Fig. 2. We replace *r*=(*β*_2_/*γ*_2_)/(*β*_1_/*γ*_1_) in (26) with *r*^*α*^, where we term *α* the *selection exponent*, which is predicted to be 1 for homogeneous networks. This gives,


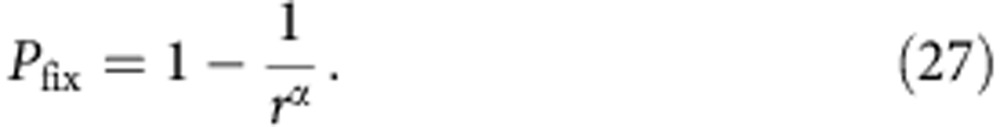


This idea is inspired by work on the simpler Moran process, where an analytic approximation demonstrates that *r* becomes *r*^*α*^ in structured populations^10^.

We fit data to this function to determine *α*, using nonlinear least squares from the nls package in R (ref. 52). The function fit well to most networks and confidence intervals on *α* were too narrow to be visible on the graphs. Supplementary Table 1 reports the *α*-values, confidence intervals and sum of the squared error for the fits.

## Author contributions

G.E.L., A.L.H. and S.B. conceived the project; G.E.L. and A.L.H. performed the simulations and calculations; all authors interpreted the results and produced the final manuscript.

## Additional information

**How to cite this article**: Leventhal, G. E. *et al*. Evolution and emergence of infectious diseases in theoretical and real-world networks. *Nat. Commun.* 6:6101 doi: 10.1038/ncomms7101 (2015).

## Supplementary Material

Supplementary Information Supplementary Figure 1 and Supplementary Table 1.

## Figures and Tables

**Table 1:** Summary statistics for networks used in Fig. 2.

**Type**	**Network**	**Source**	***N***	**‹*****k*****›**	***σ***_***k***_	***φ***
Empirical	Social (FHS)	36,37	5,253	6.5	6.8	0.68
	School	38	740	6.5*	3.3	0.04
	Hospital	39	68	6.5*	5.3	0.29
	Sexual (NATSAL)	40,41	7,578†	2.7	4.9	0.002
Theoretical	Uniform	N/A	10^4^	4	0	≈10^−4^
	Random	Erdös-Rényí/Gilbert^33^,^34^	10^4^	4	2	≈10^−4^
	Scale-free	Barabasi-Albert^35^	10^4^	4	5.3	≈10^−3^
	Small-world	Santos *et al*.^22^	10^4^	4	0	0.49

Four previously published network data sets were used along with four computationally generated networks. Reported characteristics include network size *N*, average degree ‹*k*›, s.d. in degree *σ*_*k*_ and clustering coefficient *φ*. Further details on data sets and algorithms are given in the Methods.

^*^For the school and hospital networks, a static unweighted network was sampled from the data set, which allowed ‹*k*› to be defined (to match the FHS network) and resulted in *N* being slightly smaller than the full study population to ensure a fully connected network.

^†^For the sexual network, only the degree distribution was available, which we fit to a power law function and used to construct a scale-free network with random attachment.
