# Supplementary material for: Evolution and emergence of infectious diseases in theoretical and real-world networks
Source: Nat Commun. 2015 Jan 16;6:6101. doi: 10.1038/ncomms7101 (PMC4335509; doi:10.1038/ncomms7101)
Supplement: Supplementary Information — Supplementary Figure 1 and Supplementary Table 1. [file ncomms7101-s1.pdf]

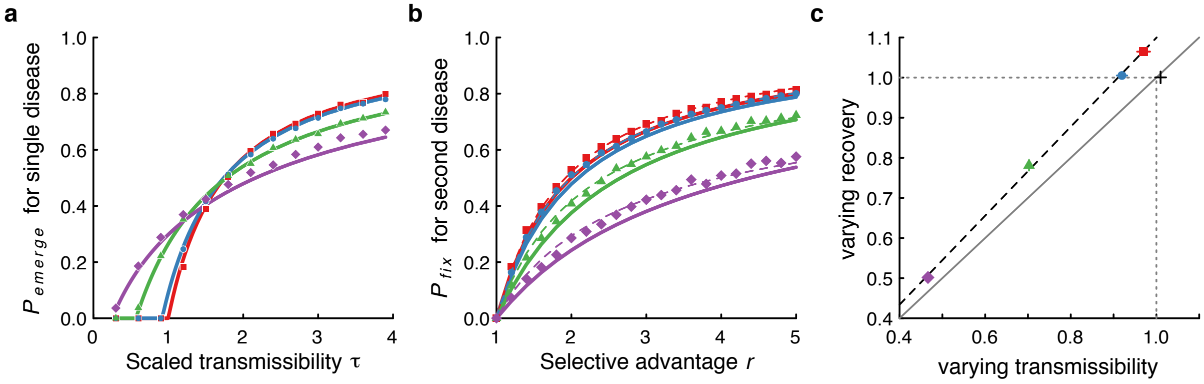


Supplementary Figure 1: **Dynamics of disease evolution in heterogeneous networks when $\gamma$ is varied compared to when $\beta$ is varied.** a) The probability that a single disease causes an epidemic (the emergence probability, $P_{\text{emerge}}$) versus the scaled transmissibility, $\tau = \beta(\langle k \rangle - 1)/\gamma$. The analytical predictions for $P_{\text{emerge}}$ also correspond well to simulations where $\gamma$ is varied (see also Figure 3). b) The probability of fixation, $P_{\text{fix}}$, versus the selective advantage of a new disease variant, $r = \gamma_2/\gamma_1$. The same as when $\beta_2$ is varied, $P_{\text{fix}}$ decreases with increasing variance in the degree distribution. c) Comparison of the selection exponent in when varying either the transmission rate $\beta_2$ or the recovery rate $\gamma_2$. Increasing variance in the degree distribution decreases the selection exponent for both varying the transmission and recovery rate, but the selection exponent is consistently higher when varying the recovery rate. The thin lines around the shapes indicate the confidence interval for the selection exponent. The black cross shows the estimate and confidence intervals of the selection exponent when varying recovery or transmission in a well-mixed population.

Supplementary Table 1: **Summary statistics for calculating the selection exponent from Equation 27 for networks used in Figure 2**.

| Type | Network | $\alpha$ | CI | SSE |
|---|---|---|---|---|
| Empirical | Social | 0.678 | [0.670,0.687] | 0.002 |
| | School | 0.810 | [0.806,0.815] | 0.001 |
| | Hospital | 0.673 | [0.666,0.680] | 0.001 |
| | Sexual | 0.343 | [0.335,0.351] | 0.004 |
| Theoretical | Random | 0.763 | [0.751,0.775] | 0.003 |
| | Scale free | 0.536 | [0.526,0.545] | 0.003 |
| | Uniform | 0.836 | [0.819,0.853] | 0.004 |
| | Small-world | 0.848 | [0.829,0.868] | 0.005 |

CI: 95 % Confidence Intervals. SSE: Sum of squared errors of fit versus data.
